# Mesoporous Mo-doped NiCo_2_O_4_ nanocrystals for enhanced electrochemical kinetics in high-performance lithium-ion batteries†

**DOI:** 10.1039/d5ra00918a

**Published:** 2025-04-28

**Authors:** Zahid Abbas, Tanveer Hussain Bokhari, Zohaib Rana, Saman Ijaz, Eman Gul, Amina Zafar, Saqib Javaid, Maria Gul, Khan Maaz, Shafqat Karim, Guolei Xiang, Mashkoor Ahmad, Amjad Nisar

**Affiliations:** a Nanomaterials Research Group, PD, PINSTECH Islamabad 44000 Pakistan chempk@gmail.com mashkoorahmad2003@yahoo.com; b Department of Chemistry, GC University Faisalabad 38000 Pakistan; c State Key Laboratory of Chemical Resource Engineering, Beijing University of Chemical Technology Beijing 100029 PR China xianggl@mail.buct.edu.cn; d Institute of Chemical Sciences, University of Peshawar Peshawar 25000 Pakistan; e CAFD, PINSTECH Islamabad 44000 Pakistan; f Theoretical Physics Division, PINSTECH Islamabad 44000 Pakistan; g MFMG, PD, PINSTECH Islamabad 44000 Pakistan

## Abstract

Capacity fading at high rates and a reduced cyclic life due to the deterioration of electrode integrity is one of the major problems in the practical applications of lithium-ion batteries. In this regard, the development of efficient and innovative electrode materials with outstanding transport features and electrochemical properties is urgently needed. In this work, mesoporous Mo-doped NiCo_2_O_4_ nanocrystals with enhanced electrochemical kinetics were prepared and investigated as an anode material for lithium-ion batteries. Experimental and density functional theory results demonstrated an increase in the specific surface area, creation of defects and enhanced conductivity. These promising features provide an opportunity to boost the lithium-storage capability of Mo-doped NiCo_2_O_4_ nanocrystals. The assembled Mo-doped NiCo_2_O_4_ electrode delivered a high initial discharge capacity of 1225 mA h g^−1^ at 50 mA g^−1^ and an excellent reversible capacity of ∼512 mA h g^−1^ at 300 mA g^−1^ with a coulombic efficiency of about 98%. Moreover, the electrode demonstrated high cyclic stability even after 300 cycles and superior rate performance compared with previously reported electrodes. These results prove that the electrochemically boosted Mo-doped NiCo_2_O_4_ structure could be an emerging electrode material for future high-performance batteries.

## Abstract 2

Mo-doped NiCo_2_O_4_ nanocrystals enhance electrochemical performance as lithium-ion battery anode. Mo incorporation introduces abundant active sites, high porosity, and structural defects, facilitating rapid Li^+^ transport and improved cycling stability.

## Graphical abstract

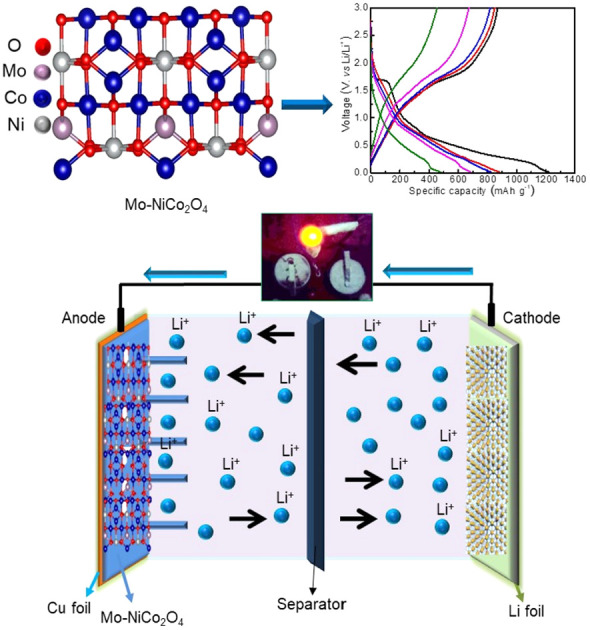

## 1 Introduction

Lithium-ion batteries (LIBs) are widely used in portable electronic devices to fulfill the current renewable, economic, and environmental demands owing to their obvious advantages compared with conventional rechargeable batteries, such as lead-acid, Ni–Cd, Ni-MH, and alkaline batteries.^1^ Most commercially available LIBs use graphite as the anode material with a specific capacity of 372 mA h g^−1^; however, it is not sufficient to fulfill the needs of next-generation electronic devices because of its limited specific capacity and cyclic performance.^2^ For the last two decades, transition metal oxides (TMOs) have been excessively explored as electrode materials for Li-ion storage owing to their superior electrochemical properties compared with those of graphite. These metal oxides include TiO_2_,^3^ MnO_2_,^4^ FeO,^5^ CoO, and spinel binary metal oxides with the formula AB_2_O_4_ (such as NiMn_2_O_4_, ZnCo_2_O_4_,^6^ CoMn_2_O_4_,^7^ ZnFe_2_O_4_,^8^ CuCo_2_O_4_,^9^ ZnCo_2_O_4_,^10^ FeCo_2_O_4_,^11^ NiCo_2_O_4_,^12,13^ ZnMn_2_O_4_,^14^ and MnCo_2_O_4_ (ref. 15)). The use of binary metal oxides is significant because of their synergistic effects, which improve electronic conductivity and reversible capacity owing to their multiple oxidation states and redox reactions.

Among the various TMOs, nickel cobalt oxide (NiCo_2_O_4_) is considered one of the most promising alternative anodes owing to its higher specific capacity (890 mA h g^−1^), better conductivity, lower diffusion resistance, multiple oxidation states, and favorable electrical conductivity.^16^ NiCo_2_O_4_ is a hole-rich p-type semiconductor that presents a large surface area to electrolyte species for efficient diffusion of Li ions.^13^ It consists of a spinel cubic structure, where the nickel cations occupy octahedral sites and the cobalt cations are randomly distributed over the tetrahedral and octahedral sites. The redox couples Ni^3+^/Ni^2+^ and Co^3+^/Co^2+^ provide it with remarkable electrocatalytic properties.^17^ Therefore, NiCo_2_O_4_ is expected to show better catalytic properties than single-component metal oxides considering its rich redox reactions caused by the presence of Ni and Co cations. Recently, various strategies have been applied to develop NiCo_2_O_4_-based advanced nanostructures for energy-storage applications, such as doping with transition metals,^18–20^ fabricating binary nanocomposites^21,22^ and controlling morphology, such as nanowires, nanorods, mesoporous nanosheets and nanoflakes.^23–26^ Liu *et al.* developed mesoporous NiCo_2_O_4_ nanoneedles on 3D graphene for batteries and supercapacitors.^27^ Abouali *et al.* reported NiCo_2_O_4_/carbon bi-functional nanotube composites and utilized these for energy-storage applications.^28^ The carbon nanotubes (CNTs) inhibited the agglomeration of the oxides and exhibited good electrochemical performance with a specific capacity of 1020 mA h g^−1^ after 200 cycles. Kong *et al.* reported needle-like NiCo_2_O_4_/polypyrrole (PPy) nanowires on carbon cloth (CC) synthesized by a hydrothermal method and chemical oxidative polymerization.^29^ However, the large volume expansion/contraction, particle aggregation, detachment from the substrate during lithiation/delithiation processes, rapid capacity fading and reduction in the kinetics of ion diffusion hinder the application of these materials in high-performance LIBs.^30^ An effective strategy to ameliorate these drawbacks is to dope NiCo_2_O_4_ with different transition metals, such as Al, N, S, Zn, Fe, W, or P.^20,31–36^ However, there are huge challenge in obtaining admirable energy density owing to structural instability and chemical incompatibility.

Molybdenum is an appealing candidate for doping due to its versatile oxidation states and excellent redox properties.^19,37^ Also, its comparable ionic radius to cobalt makes it a perfect choice as a dopant that could be included without disturbing the NCO crystal structural stability. Furthermore, the option for optimization of the Gibbs free energy for electrocatalytic reactions, enhancement in pore concentration, as well as the creation of oxygen vacancies make it an ideal dopant to use to synthesize novel nanocrystals for Li-ion storage.^18,32^ Herein, we report the synthesis of mesoporous Mo-doped NiCo_2_O_4_ nanocrystals with tuned morphology and chemical composition *via* a facile solvothermal approach. An electrode developed using this material exhibits high specific capacity, excellent cyclic stability and high rate capability. The enhanced electrochemical performance may be due to the presence of more redox sites, higher electrical conductivity, and fast electron and lithium-ion transportation during the charge–discharge cycling process.

## 2 Experimental

### 2.1 Chemical reagents

Nickel nitrate hexahydrate (Ni(NO_3_)_2_·6H_2_O, ∼99%), and cobalt nitrate hexahydrate (Co(NO_3_)_2_·6H_2_O, ∼99%) were purchased from Scharlau. Sodium hydroxide (NaOH, ∼99%), *N*,*N*-dimethylformamide (DMF C_3_H_7_NO, ∼98%), glycerol (C_3_H_8_O_3_, ∼98%) and phthalic anhydride (C_8_H_4_O_3_, ∼99%) were purchased from Merck. Ammonium molybdate tetrahydrate ((NH_4_)_6_Mo_7_O_24_·4H_2_O, ∼98%) was purchased from BDH. All these reagents were of analytical grade and used without any further purification.

### 2.2 Synthesis of NiCo_2_O_4_ nanoparticles

NiCo_2_O_4_ nanoparticles (NCO) were synthesized by a hydrothermal method. In a typical synthesis, 0.193 g (0.6 mmol) of Ni(NO_3_)_2_·6H_2_O and 0.38 g (1.3 mmol) of Co(NO_3_)_2_·6H_2_O were dissolved in 30 mL deionized water followed by magnetic stirring for 30 min to obtain a homogenous solution. Subsequently, NaOH (0.3 M) 22 mL aqueous solution was poured into the above solution and stirring was continued for a further 15 min. The whole precursor mixture was then transferred into a 50 mL Teflon-lined stainless-steel autoclave to react at 100 °C overnight. After cooling to room temperature, the sample was washed three times with deionized water and ethanol following centrifugation. The obtained precipitate was dried at 60 °C for 6 h, followed by calcination at 250 °C for 7 h to obtain the final product.

### 2.3 Synthesis of Mo-doped NiCo_2_O_4_ nanocrystals

Mo-doped NiCo_2_O_4_ nanocrystals (Mo-NCO) were synthesized using a solvothermal approach. In a typical synthesis, 0.174 g (0.6 mmol) of Ni(NO_3_)_2_·6H_2_O, 0.348 g (1.2 mmol) of Co(NO_3_)_2_·6H_2_O and 0.74 g (0.6 mmol) of (NH_4_)_6_Mo_7_O_24_·4H_2_O were dispersed in 30 mL DMF–glycerol mixed solvent solution (v/v: 2 : 1) using ultrasonic dispersion and magnetic stirring for 30 min. Afterwards, 0.354 g (2.4 mmol) of phthalic anhydride was added in to the dispersion under continuous stirring to obtain a homogeneous solution. The obtained mixture was transferred into a 50 mL Teflon-lined stainless-steel autoclave and heated at 180 °C for 6 h. Afterwards, the autoclave was cooled to room temperature by a natural convection process and the resulting brown precipitate was washed twice with DMF and ethanol, respectively, following centrifugation. The collected precipitates were dried at 80 °C overnight followed by calcination at 500 °C for 1 h in a box furnace to obtain the final product.

### 2.4 Material characterization

X-Ray diffraction (XRD) patterns of the obtained products were collected on a Rigaku D/Max-2500 instrument equipped with Cu Kα radiation (*λ* = 1.5406 Å) over the 2*θ* range of 10°–80°. FTIR spectra were collected using a Thermo Fisher Scientific Nicolet™ iS50 FTIR spectrometer. The morphology and structure of the obtained products were characterized by scanning electron microscopy (SEM) using a TESCAN MIRA A-3 system equipped with an energy-dispersive X-ray spectroscopy (EDS) unit. The composition and elemental distribution were studied by EDS analysis. High-resolution transmission electron microscopy (HRTEM) and elemental mapping were performed using a JEOL JEM-2100F system at 200 kV. X-Ray photoelectron spectroscopy (XPS) analysis was conducted with a Thermo Fisher Scientific ESCALAB 250Xi instrument with a monochromatic Al Kα radiation source to characterize the surface composition and chemical states of the obtained nanocrystals. Adsorption–desorption isotherms were measured using a Quantachrome Instruments gas sorption analyzer (version 5.23) at 77 K with an initial degas treatment at 373 K. Nitrogen was used as the adsorbate for the pore size and surface area measurements of the obtained Mo-NCO samples.

### 2.5 Coin cell assembly and electrochemical measurements

The electrochemical performances of the obtained materials were measured using a CR2025 coin cell, which was assembled in an argon-filled glove box with a Li-metal chip as the counter electrode. For preparing the working electrode, 70 wt% active material, 20 wt% carbon black, and 10 wt% polyvinylidene fluoride (PVDF) were mixed into an appropriate volume of *N*-methyl pyrrolidone (NMP) as the solvent. The mixture was stirred for 24 h to attain a homogeneous slurry. The slurry was then uniformly coated on Cu foil, followed by overnight drying in a vacuum oven. LiPF_6_ (1 M) solution in ethylene carbonate (EC), diethyl carbonate (DEC), and dimethyl carbonate (DMC) (4 : 2 : 4 by volume) was used as the electrolyte. Celgard-2400 film was used as the separator. Galvanostatic discharge/charge measurements were performed in the voltage range of 0.01–3.0 V (*vs.* Li^+^/Li) using an MTI-BSTA-MA battery testing system. Cyclic voltammetry (CV) measurements of the battery were carried out using a CHI660E (Chenhua Shanghai, China) electrochemical workstation at a scan rate of 0.5 mV s^−1^. Electrochemical impedance spectroscopy (EIS) was conducted in the frequency range of 0.01 Hz to 100 kHz with a 5 mV amplitude.

### 2.6 DFT calculations

All the DFT calculations were performed employing the plane-wave pseudopotential approach as implemented in VASP code. For the exchange-correlation functional, the generalized gradient approximation (GGA) within the Perdew–Burke–Ernzerhof (PBE) parameterization was used.^38^ Modeling of NiCo_2_O_4_ was performed by adopting a cubic spinel structure with the *Fd*3*m* space group with initial lattice parameters of *a* = *b* = *c* = 8.10 Å. During the structural optimization, both the atomic positions and lattice parameters were relaxed. Herein, we employed an inverse spinel structure, which has been shown to be energetically favorable (see Fig. 7a).^39–41^ To simulate the impact of Mo-doping, one transition metal 3d atom within NiCo_2_O_4_ was replaced by Mo, corresponding to ∼5% doping. Considering the presence of open shell atoms within the system, spin-polarized calculations were performed. A kinetic energy cutoff of 400 eV was used, while a 2 × 2 × 2 *k*-mesh was employed for Brillouin zone sampling. The convergence criteria for the total energy and forces were set to 10^−5^ eV and 0.01 eV Å^−1^, respectively. In the absence of a reliable phase diagram, formation energies were calculated with respect to the ground state energies of the constituent elements, *e.g.*, half of the O_2_ energy was used for oxygen.

## 3 Results and discussion

**Fig. 1:**
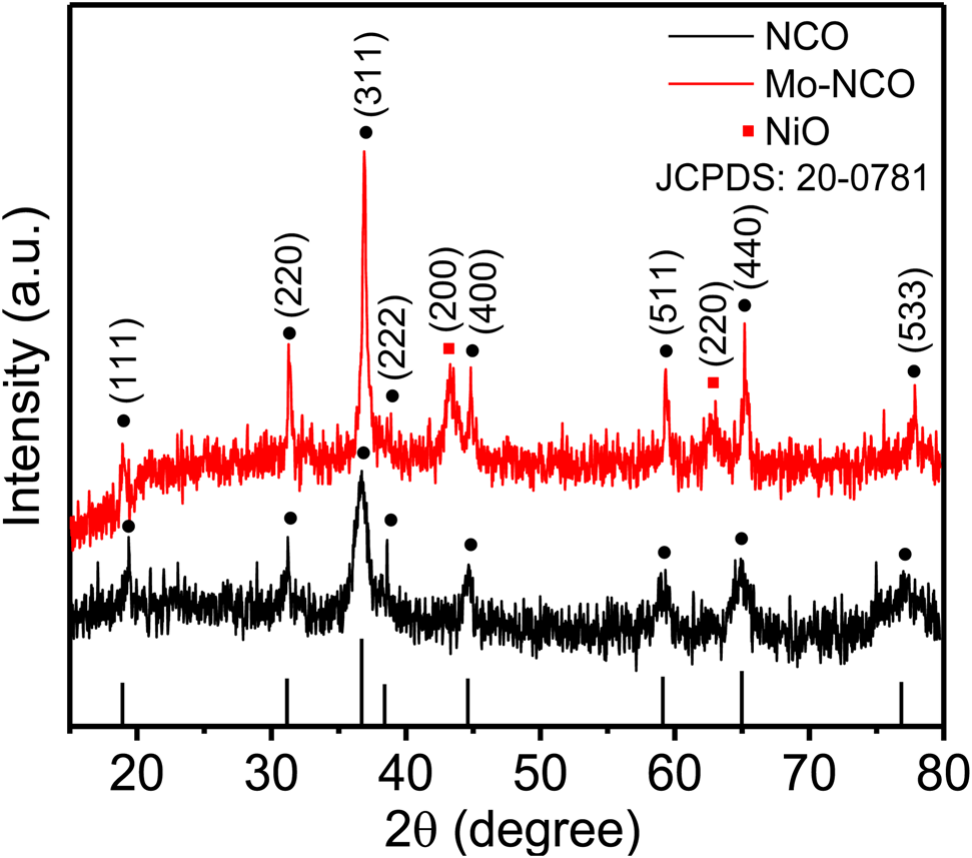
XRD patterns of NCO and Mo-NCO nanocrystals.

Mesoporous Mo-doped nickel cobalt oxide nanocrystals were synthesized by a facile one-step hydrothermal process, as illustrated in Fig. S1† (ESI). XRD measurements were performed to evaluate the phase and crystal structure of the synthesized materials. Fig. 1 shows the XRD patterns of NCO and Mo-NCO. The crystal phase of NCO was confirmed by the five major peaks observed at 31.20°, 36.70°, 44.65°, 59.29° and 64.95°, corresponding to the (220), (311), (400), (511) and (440) planes, and the three minor peaks at 18.90°, 38.60°, 77.52°, corresponding to the (111), (222), (533) planes, respectively, revealing the spinel cubic structure (JCPDS card 20-0781). The XRD measurements of Mo-NCO exhibited a similar pattern, but with three additional peaks at 37.23°, 43.29°, and 63.01°, which could be indexed to the (200), (111) and (220) planes of cubic nickel oxide (JCPDS card 47-1049). Notably, the intensity of Mo-NCO was higher than that of pristine NCO, which could be attributed to the enhanced crystallinity and preferential growth along specific planes ((311), (400), and (440)), resulting in increased peak intensities. Additionally, the magnified plot of the dominant XRD peaks of Mo-NCO ((220), (311), (400)) showed a slight shift toward higher 2*θ* values (Fig. S2†), likely due to the incorporation of Mo in the lattice, resulting in compressive strain. ^18,42^ The radius of Mo^6+^ ions (0.055 nm) was smaller than Co^3+^ (0.068 nm); therefore, the partial substitution of Co^3+^ ions by Mo^6+^ ions in the lattice of NCO led to a lattice contraction, reduced *d*-spacing, defect formation, and increased exposure of the interfacial active sites, thereby enhancing the conductivity and reaction kinetics.

The FTIR spectra of the NCO and Mo-NCO nanocrystals are shown in Fig. S3.† The sharp peaks at 535 and 628 cm^−1^ could be attributed to Ni–O and Co–O stretching vibrations, respectively. The broad band located at 1623 cm^−1^ in both NCO and Mo-NCO nanocrystals corresponded to the bending modes of H_2_O molecules;^43,44^ while the peak at 1385 cm^−1^ in Mo-NCO was due to the adsorption of CO_2_ in the air. The peaks of Ni and Co were present at nearly the same position, but with a slight shift in the peak position (Fig. S4†) toward a lower wavenumber, which may be attributed to the dopant material.

**Fig. 2:**
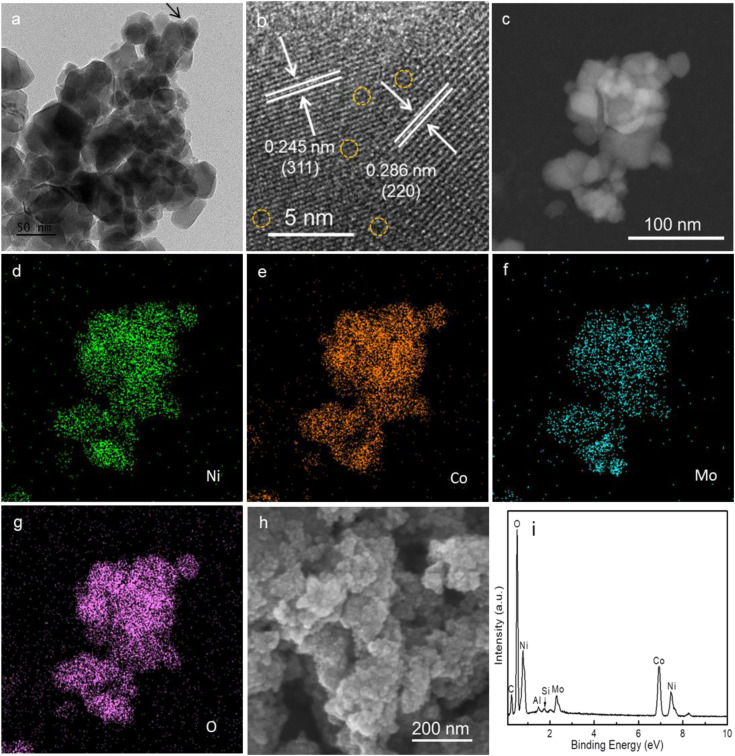
(a) and (b) TEM and HRTEM images of the Mo-NCO nanocrystals. (c) STEM image of the Mo-NCO nanocrystals. (d)–(g) Elemental mapping exhibiting the presence of Ni, Co, Mo, and O. (h) SEM image of the Mo-NCO nanocrystals. (i) EDS spectrum of Mo-NCO.

TEM analysis demonstrated that the Mo-NCO was composed of distinct nanocrystals with sizes ranging from 20 to 40 nm, as shown in Fig. 2a. The HRTEM images confirmed the spinel cubic crystal structure of Mo-NCO nanocrystals by depicting the characteristic lattice spacings of 0.245 and 0.286 nm corresponding to the (311) and (220) planes, respectively (Fig. 2b).^45^ Moreover, the high-resolution images revealed the formation of native point defects, which may provide conducting paths for ion movements and enhanced electrochemical properties for energy storage.^46–48^Fig. 2c–g present the STEM image and elemental mapping results, confirming the even distribution of Ni, Co, Mo, and O on the surface of Mo-NCO nanocrystals and verifying the Mo-doping in NCO. The SEM images of NCO and Mo-NCO displayed a similar porous morphology as observed in the TEM investigations (Fig. 2h and S5†). EDS analysis coupled with SEM further supported the TEM and STEM results, confirming the successful syntheses of NCO and Mo-NCO nanocrystals (Fig. 2i and S6, ESI†) without any impurities. Additionally, a Mo-doping level of approximately 5% by weight was noted.

**Fig. 3:**
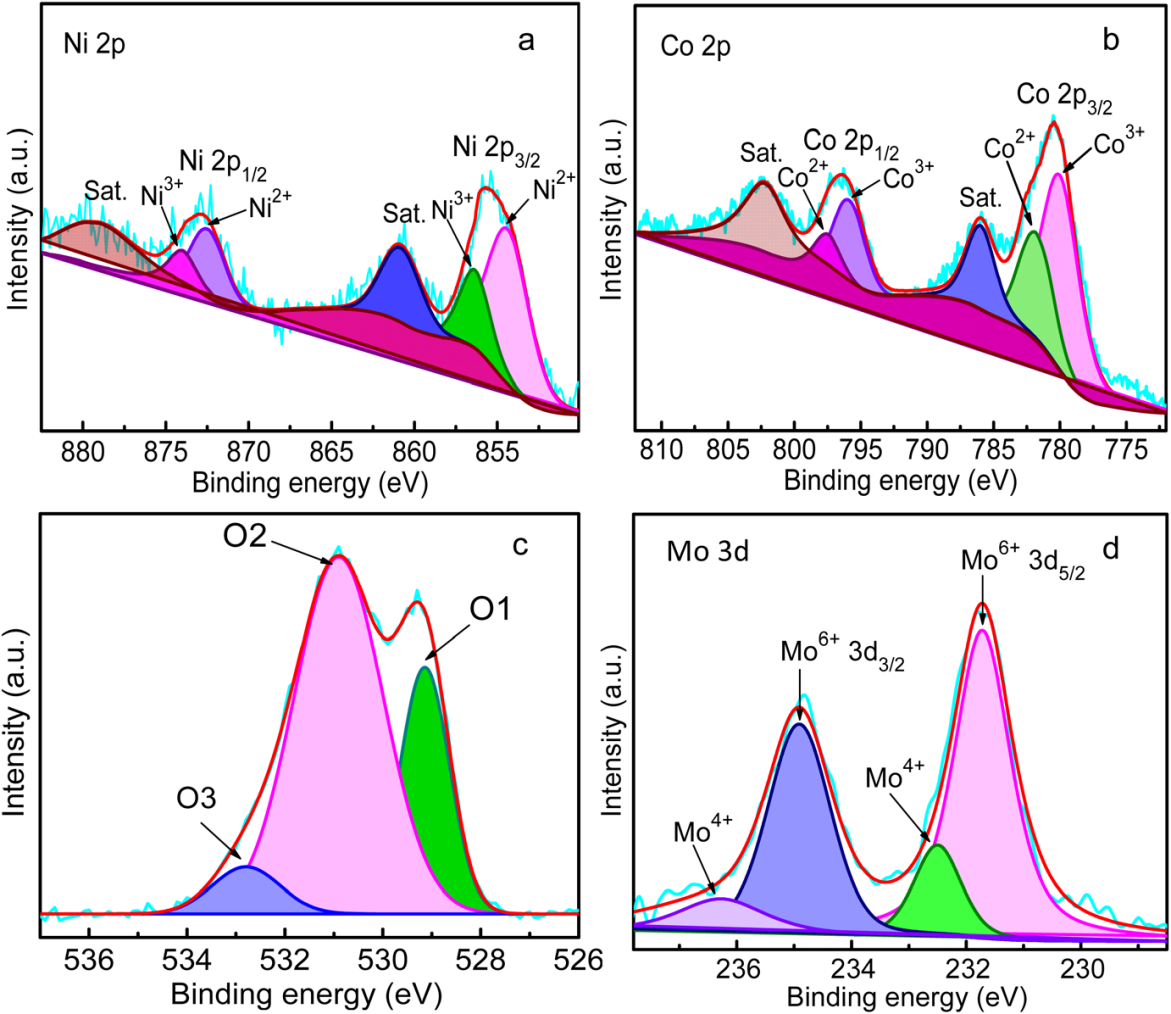
XPS spectrum of Mo-NCO nanocrystals: narrow scans of Ni 2p (a) Co 2p (b) O 1s (c) and Mo 3d (d).

To evaluate the chemical composition and valence state of the nanocrystals, XPS measurements were performed, as shown in Fig. 3. In the survey spectrum of Mo-NCO, peaks could be observed located at 855, 780, 529, and 230 eV, attributed to Ni 2p, Co 2p, O 1s and Mo 3d (Fig. S7†), confirming the successful doping of Mo in the nickel cobalt oxide lattice. Fig. 3a displays the high-resolution XPS spectrum of Ni 2p, exhibiting a spin–orbit doublet peak pair, *i.e.*, Ni 2p_3/2_ and Ni 2p_1/2_, at 855.7 and 872.9 eV, respectively, with two associated satellite peaks at 861.5 and 879.4 eV. The peak at 855.7 eV could be deconvoluted into two peaks centered at 854.5 and 856.4 eV, assigned to Ni^3+^ and Ni^2+^, respectively. Similarly, the peak at 872.9 eV was deconvoluted into two peaks centered at 872.4 and 874.1 eV, assigned to Ni^3+^ and Ni^2+^, respectively.^49–52^ The narrow scan spectrum of Co 2p displayed two doublets, namely Co 2p_3/2_ (780.4 eV) and Co 2p_1/2_ (796.4 eV), with two satellite peaks located at 786.0 and 802.3 eV (Fig. 3b). The deconvoluted peaks at 780.1 and 795.9 eV represented Co^3+^, while the peaks at 781.9 and 797.5 eV were ascribed to Co^2+^.^50,52^Fig. 3c shows the high-resolution spectrum for O1s, which was deconvoluted into three peaks at 529.1 (O1), 530.9 (O2) and 532.8 eV (O3). The peak at 529.1 eV corresponded to lattice oxygen, while that at 530.9 eV corresponded to oxygen vacancies or point defects. The small peak that appeared at 532.8 eV was associated with chemical/physical adsorbed water molecules on the surface of Mo-NCO. Moreover, the high intensity of the O2 peak suggested an increased number of oxygen vacancies due to Mo-doping.^51,53^ The higher oxygen vacancies can not only enhance the electrical conductivity but also enhance the charge-transfer kinetics of the Mo-NCO structure, indicating it is suitable for Li-ion storage.^18^Fig. 3d shows the Mo 3d spectrum of Mo-NCO. The peaks located at 231.7 and 234.9 eV were attributed to Mo^6+^ 3d_5/2_ and Mo^6+^ 3d_3/2_, respectively, while the two peaks at binding energies of 232.5 and 236.2 eV were assigned to Mo^4+^ 3d_5/2_ and Mo^4+^ 3d_3/2_, respectively. From the Mo 3d spectrum, it is evident that the molybdenum doped in the nickel cobalt oxide lattice was predominantly in the form of the high valence state Mo^6+^.^54^ The doping of high oxidation state metal ions in binary metal oxides is a promising strategy to engineer point defects, and to enhance the conductivity and electrochemical properties of spinel structures, as reported earlier.^37^ These features are highly significant for efficient and reversible energy storage in LIBs.

**Fig. 4:**
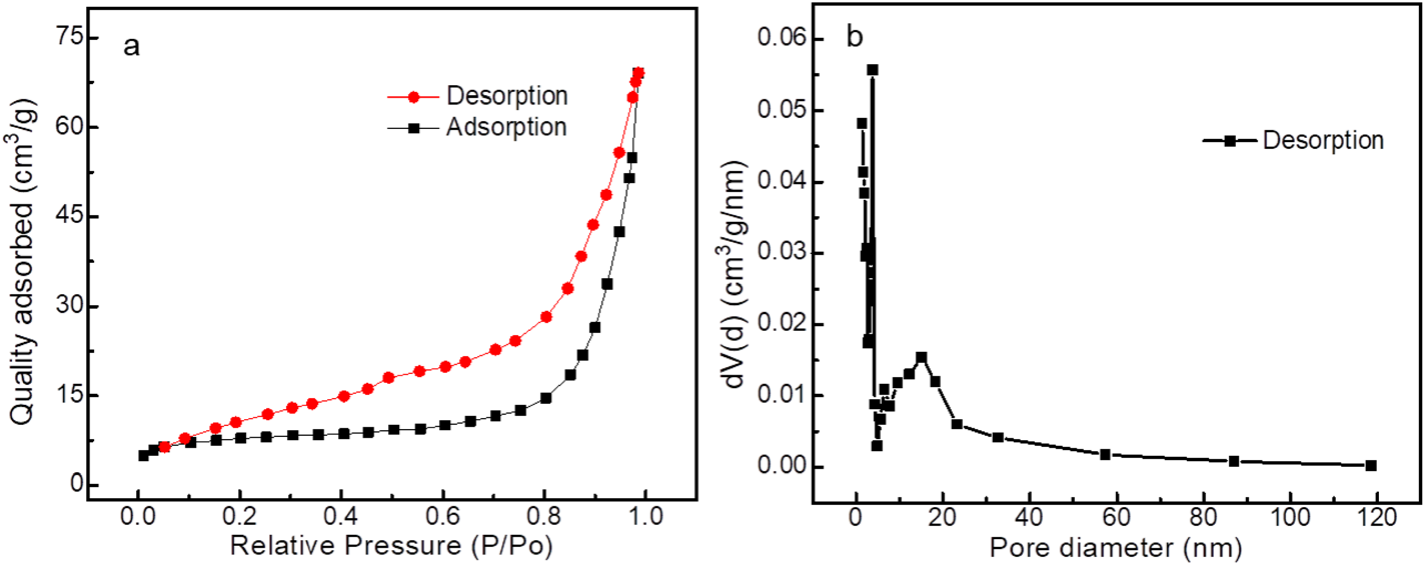
Nitrogen adsorption/desorption isotherms of Mo-NCO nanocrystals (a). Pore-size distribution (b).

In order to investigate the specific surface area and porous nature of the Mo-NCO sample, nitrogen adsorption/desorption measurements were performed at 77 K. As evident from Fig. 4a, the curve belonged to a type IV isotherm with an H1 hysteresis loop, indicating the mesoporous characteristic nature of Mo-NCO, which was consistent with the TEM results. Using the Brunauer–Emmett–Teller model, the surface area of Mo-NCO was calculated as 70 m^2^ g^−1^ with a total pore volume of 0.15 cm^3^ g^−1^. According to the Barrett–Joyner–Halenda (BJH) model, the pore-size distribution of Mo-NCO nanocrystals showed two maxima at 3.79 and 15.21 nm (Fig. 4b), which further confirmed the mesoporous nature of the sample.^55,56^ Obviously, the mesoporous nanocrystals with a large surface area and appropriate pore channels can facilitate the diffusion of Li ions during the charge–discharge phenomenon and provide enough room for accommodating volume changes.

**Fig. 5:**
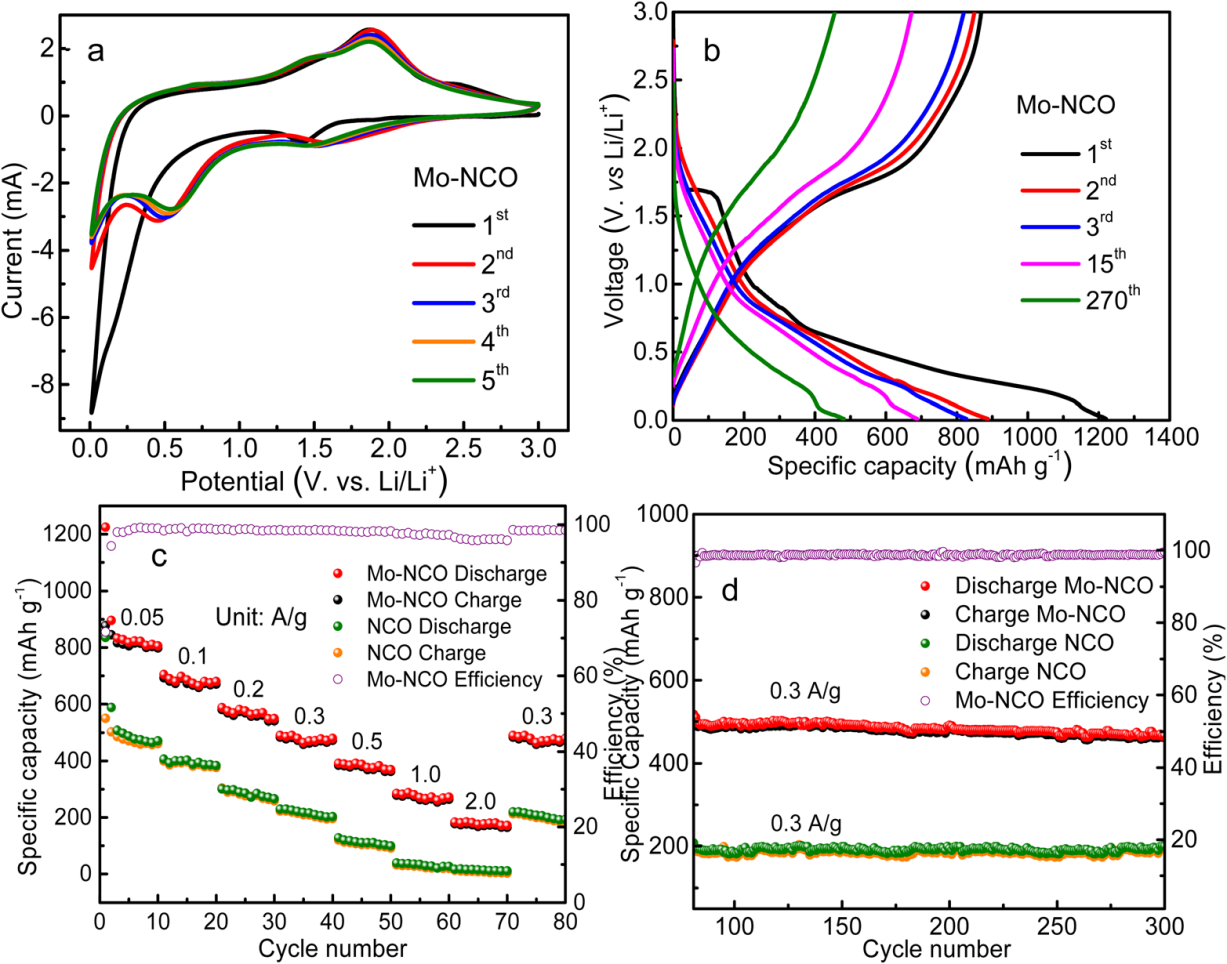
(a) CV curves of the 1st, 2nd, 3rd and 4th cycles of the Mo-NCO electrode. (b) Galvanostatic charge/discharge voltage profiles for the 1st, 2nd, 3rd, 11th, and 270th cycle of the Mo-NCO electrode. (c) Rate performance of the NCO and Mo-NCO electrodes at different current rates in the voltage range of 0.01–3.0 V *vs.* (Li/Li^+^), and efficiency of Mo-NCO. (d) Cyclic performances of the NCO and Mo-NCO electrodes at a current density of 300 mA g^−1^ up to 300th cycle, and efficiency of Mo-NCO.

The reactivity of the Mo-NCO electrode was studied by cyclic voltammetry. Fig. 5a shows the CV curves of the as-prepared Mo-NCO anode *versus* Li/Li^+^ at a scan rate of 0.5 mV s^−1^ in the voltage range of 0.01–3.0 V. In the first cycle, a strong cathodic peak with a shoulder at 0.17 V was observed, ascribed to the decomposition of the electrolyte and formation of the solid electrolyte interface (SEI), while the peak centered at 1.42 V and four weak peaks centered at 2.68, 2.30, 2.14 and 1.88 V (zoomed-in view, Fig. S8†) could be attributed to the reduction of nickel cobalt oxide to metallic Ni, Co and the formation of Li_2_O, following eqn (1), as well as certain irreversible reactions. In the following first anodic scan, two peaks were observed centered at 1.44 and 1.87 V, assigned to the oxidation of metallic Ni and Co to NiO, CoO and Co_3_O_4_ (eqn (2)–(4)), while the peak at 2.45 V was assigned to the partial decomposition of SEI.^16,52^ In the 2nd cycle, the cathodic peak centered at 1.61 V was attributed to the alloying of CoO with Li^+^, while the peak centered at 0.45 was attributed to the alloying of NiO with Li^+^ ions. The anodic peaks at 1.44 and 1.88 V were ascribed to the de-alloying process of Ni and Co, respectively. In the subsequent three cycles, the cathodic and anodic peaks exhibited small shifts from the original positions, which may be due to disturbances in the crystal structure during the lithiation and delithiation processes (Fig. S9†). Following the 2nd cycle, all the CV curves well overlapped, indicating the good electrochemical reversibility and stability of the Mo-NCO electrode.

1 NiCo_2_O_4_ + 8Li^+^ + 8e^−^ → 2Co + Ni + 4Li_2_O

2 Ni + Li_2_O ↔ NiO + 2Li^+^ + 2e^−^

3 Co + Li_2_O ↔ CoO + 2Li^+^ + 2e^−^

4 CoO + 1/3Li_2_O ↔ 1/3Co_3_O_4_ + 2/3Li^+^ + 2/3e^−^

Fig. 5b shows the galvanostatic charge–discharge profiles of the Mo-NCO nanocrystals for the 1st, 2nd, 3rd, 11th (at a current density of 0.05 A g^−1^) and 270th (at a current density of 0.3 A g^−1^) cycles in the voltage range of 0.01–3.0 V with discharge capacities of 1225, 895, 832, 727 and 485 mA h g^−1^. For the first cycle, the efficiency of the Mo-NCO electrode was found to be about 71%. The irreversible capacity loss in the first cycle was due to the SEI layer and other side reactions. During the first discharge process, the plateau at 1.6 V indicated the conversion of NiCo_2_O_4_ to metallic Ni, Co and the formation of Li_2_O. The region from 1.6 V, with an inclined plateau starting at 0.8 V and a shoulder at 0.14 V, was due to the formation of the SEI layer, alloying (caused by Li^+^ ions insertion) and certain irreversible reactions. This behavior agreed well with the CV measurements. After the first few cycles, the coulombic efficiency of the Mo-NCO electrode reached 98%, demonstrating the high reversibility of the discharge–charge process. Fig. S10 and S11† display the CV measurements and charge–discharge profiles of NCO in the first three cycles, showing good agreement with each other. The discharge capacities of NCO were calculated to be 872, 590, 460 mA h g^−1^. Obviously, the Mo-NCO electrode exhibited a significantly enhanced specific capacity compared to the NCO electrode, which may be attributed to its different electrochemical behavior, as evident from the CV and GCD profiles.

The rate performances of the Mo-NCO and NCO electrodes were further evaluated employing different current densities. Fig. 5c shows the rate performance profiles of Mo-NCO and NCO at various current densities of 0.05, 0.1, 0.2, 0.3, 0.5, 1.0 and 2.0 A g^−1^. Clearly, the Mo-NCO electrode exhibited superior rate performance compared to NCO. For instance, at 0.05, 0.2, 0.5 and 2 A g^−1^ current densities, the NCO electrode delivered specific discharge capacities of 872, 416, 230 and 40 mA h g^−1^, while the Mo-NCO electrode delivered specific discharge capacities of 1225, 614, 416 and 205 mA h g^−1^, respectively. At higher current rates, the NCO electrode showed a negligible reversible capacity (Fig. 5c). For Mo-NCO, when the current density returned to 0.3 A g^−1^, its reversible capacity also returned to its original discharge value of ∼510 mA h g^−1^, which at the 270th cycle was marginally decreased to 485 mA h g^−1^. It was thus evident that the Mo-NCO electrode delivered a superior rate capability performance, which could be due to the better electronic conductivity and charge kinetics of the Mo-NCO electrode.

The cyclic performance of the Mo-NCO electrode was further explored by extending the rate performance measurements up to 300 cycles at a current density of 0.3 A g^−1^. Fig. 5d demonstrates the electrode's outstanding reversible lithium-ion-storage capability in the potential range of 0.01–3.0 V with a capacity retention of about 94% and coulombic efficiency of about 98%. The outstanding rate and cycling performance of the Mo-NCO electrode could be primarily ascribed to its enhanced spinel structure stability, stable Mo^6+^/Co^3+^ states and improved electronic conductivity due to the doping of Mo in the NiCo_2_O_4_ crystal lattice, which could facilitate the transport of electrons and lithium ions during repeated cycles. Also, the mesoporous nature and point defects of the Mo-NCO nanocrystals could efficiently accommodate volume changes and enhance the electrode stability for attaining long-life batteries. Furthermore, the reversible energy storage of the Mo-NCO electrode was superior and comparable to previously reported results related to nickel cobalt oxide-based nanostructures, as illustrated in Table S1 and Fig. S12.†

**Fig. 6:**
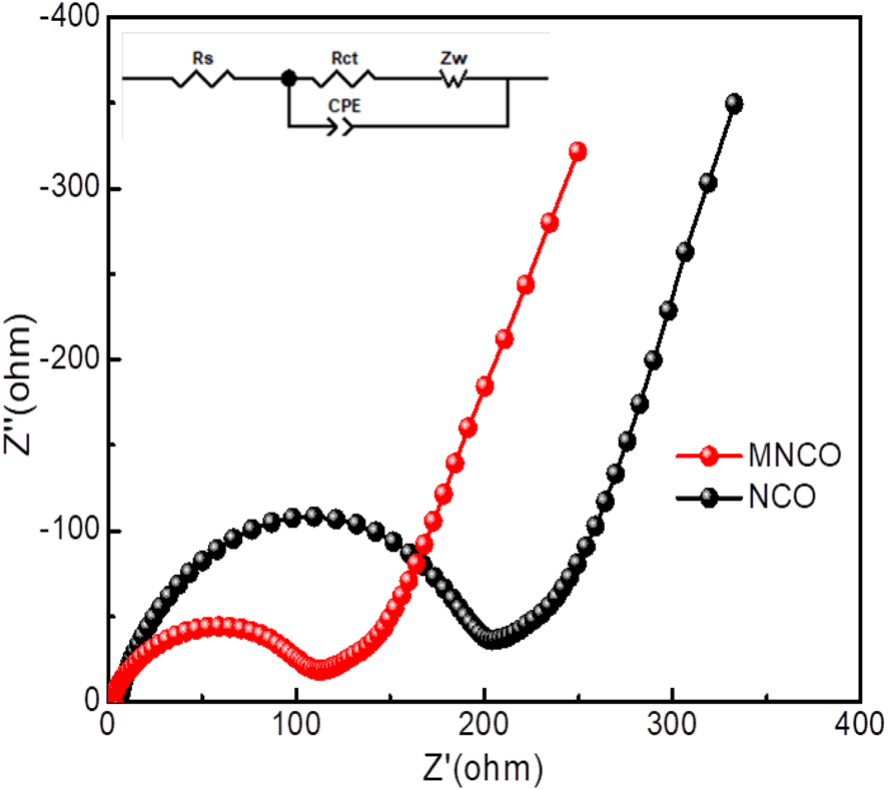
Nyquist plots of Mo-NCO and NCO with an amplitude of 5 mV over the frequency range of 100–0.01 Hz; inset shows the equivalent electric circuit used to fit the raw impedance data in order to calculate the values of *R*_s_ and *R*_ct_.

To probe the effect of molybdenum doping on the charge-transport kinetics of the mesoporous Mo-NCO electrode, electrochemical impedance spectroscopy (EIS) was conducted in the frequency range of 0.01 Hz to 100 kHz. Fig. 6 displays the Nyquist plots of the Mo-NCO and NCO electrodes. The semicircle in the high-frequency region is associated with charge-transfer resistance (*R*_ct_) while the sloping straight line (∼45°) represents the Warburg impedance (*Z*_w_) in the low-frequency region. Corresponding to lithium-ion diffusion in the cell. Clearly, both the Mo-NCO and NCO electrodes exhibited depressed semicircles, indicating non-ideal capacitive behaviors [*C* = *R*^1−*n*/*n*^(CPE)^1/*n*^]; where *C*, *R*, CPE and *n* signify the associated component's capacitance, resistance, constant phase element and departure from the ideal Debye behavior, respectively. The fitted equivalent circuit in the experimental data revealed one *RC* circuit in combination with resistance connected in series (see the inset in Fig. 6). The fitting parameters, *R*_ct_ and *R*_s_ are presented in Table S2.† The Mo-NCO electrode possessed a lower *R*_ct_ compared to NCO. Evidently, Mo-doping in the nickel cobalt oxide nanocrystals boosted the electrochemical charge-transfer kinetics and conductivity of the fabricated electrode, enabling attaining a high rate and durable Li-ion battery.

**Fig. 7:**
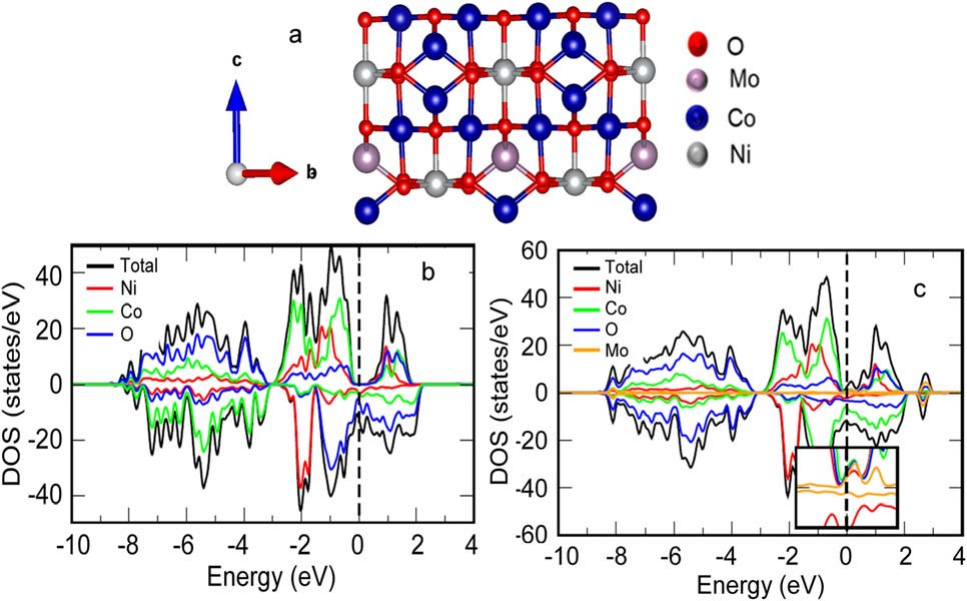
(a) Schematic depiction of the side view of the Mo-NCO crystal structure. Spin-polarized PDOS of NCO (b) and Mo-NCO (c) nanocrystals. Spin-up (-down) contributions are presented by positive (negative) DOS values. Dashed vertical line indicates the Fermi level (EF), while the inset in (c) shows the region close to the EF.

To further elucidate the electrochemical properties, the apparent Li-ion diffusion coefficient (*D*_Li^+^_) was determined by EIS analysis. The calculated *D*_Li^+^_ values for Mo-NCO and NCO were 10^−12^ and 10^−13^ cm^2^ s^−1^, respectively. Obviously, the higher value of *D*_Li^+^_ for Mo-NCO facilitated enhanced kinetics and Li-ion storage. The relevant plots and calculation details are provided in the ESI (Fig. S13†).^57^ Moreover, DFT calculations were performed to support the experimental findings. Fig. 7b and c present the partial density of states (PDOS) of NCO and Mo-NCO, which highlight the impact of Mo-doping on the electronic structure. For the pristine NCO (Fig. 7b), the electronic structure was half-metallic and a noticeable gap was observed in the spin-up channel, while the spin-down channel was conducting, as highlighted by the presence of states at the Fermi level (EF). Moreover, the ground state was ferrimagnetic, such that the magnetic moment of Ni (−0.80 μβ) was aligned opposite to that of Co (2.33 μβ), yielding a net magnetic moment of ∼15.50 μβ. Overall, these findings are in line with previous DFT calculations.^58^ Mo-doping had a subtle impact on the electronic structure (Fig. 7c); unlike pristine NCO, the gap in the spin-up channel was closed, leading to the appearance of states at and around the EF for both spins. Therefore, the transport characteristics of NCO changed from half-metallic to metallic due to Mo-doping. Typically, the redox reaction kinetics and thus the electrochemical performance of a cell are improved with the increase in conductivity of an electrode.^59^ Thus, the change in electronic structure of NCO due to the Mo-doping will have important consequences for battery performance. For pristine NCO, conductivity was possible for only one (down) spin channel. On the other hand, both spin channels were conducting for the Mo-NCO nanocrystals. As a result, Mo-doping in the NiCo_2_O_4_ anode improved the performance of the anode due to the enhanced reaction kinetics at the interface. These results further confirmed the EIS and GCD findings, wherein a much lower charge-transfer resistance was measured for Mo-NCO electrode while an increase in specific capacity of the anode was observed.

To ascertain the stability of Mo-NCO in comparison to pristine NCO, the formation energies per atom for NCO and Mo-NCO were calculated and were found to be ∼−0.758 and ∼−0.767 eV, respectively. The comparative reduction of the formation energies due to Mo-doping clearly confirmed the stability of Mo-NCO. This is indeed in line with experimental work where Mo-NCO has been successfully synthesized. ^60^

## 4 Conclusions

In summary, we successfully synthesized mesoporous Mo-doped nickel cobalt oxide nanocrystals using a facile hydrothermal approach. Mo-doping was successfully applied to tune and enhance the specific surface area, point defects and electronic conductivity of transition metal oxides. The developed Mo-doped NiCo_2_O_4_ nanocrystals electrode exhibited lower capacity fading, a superior rate capability and extended cyclic life for LIBs. These promising features of the developed material may be attributed to its mesoporous nature, enhanced electrochemical kinetics, and facile lithium-ion transport during the charge–discharge phenomenon. The applied concept could be extended to develop other novel nanomaterials with enhanced electrochemical properties for efficient energy storage for practical applications.

## Data availability

The data supporting this article have been included as part of the ESI.†

## Author contributions

Zahid Abbas: experimentation, investigation, writing – original draft, and data curation. Tanveer Hussain Bokhari: methodology, resources, and supervision. Zohaib Rana: material synthesis and investigation. Saman Ijaz: experimentation, Eman Gul: experimentation. Amina Zafar: formal analysis. Saqib Javaid: DFT calculations. Maria Gul: investigation. Khan Maaz: review, editing. Shafqat Karim: resources and formal analysis. Guolei Xiang: methodology, formal analysis, validation, and manuscript writing. Mashkoor Ahmad: investigation, methodology, formal analysis, and validation. Amjad Nisar: conceptualization, validation, formal analysis, funding acquisition, writing manuscript, supervision, and project administration.

## Conflicts of interest

There are no conflicts to declare.

## Supplementary Material

RA-015-D5RA00918A-s001
