# Supplementary material for: Mesoporous Mo-doped NiCo2O4 nanocrystals for enhanced electrochemical kinetics in high-performance lithium-ion batteries
Source: RSC Adv. 2025 Apr 28;15(17):13552–60. doi: 10.1039/d5ra00918a (PMC12035699; doi:10.1039/d5ra00918a)
Supplement: RA-015-D5RA00918A-s001 [file RA-015-D5RA00918A-s001.pdf]

Supplementary Information (SI) for RSC Advances.
This journal is © The Royal Society of Chemistry 2025

Electronic Supplementary Information

Mesoporous Mo-Doped $NiCo_2O_4$ Nanocrystals for Enhanced Electrochemical Kinetics in High-Performance Lithium-Ion Batteries

Zahid Abbas,[a,b] Tanveer Hussain Bokhari,[b] Zohaib Rana,[c] Saman Ijaz,[b] Eman Gul,[d] Amina Zafar,[e] Saqib Javaid,[f] Maria Gul,[g] Khan Maaz,[a] Shafqat Karim,[a] Guolei Xiang,*[c] Mashkoor Ahmad,*[a] Amjad Nisar*[a]

[a] Nanomaterials Research Group, PD, PINSTECH, Islamabad 44000, Pakistan

[b] Department of Chemistry, GC University, Faisalabad 38000, Pakistan

[c] State Key Laboratory of Chemical Resource Engineering, Beijing University of Chemical Technology, Beijing 100029, PR China

[d] Institute of Chemical Sciences, University of Peshawar, Peshawar 25000, Pakistan

[e] CAFD, PINSTECH, Islamabad 44000, Pakistan

[f] Theoretical Physics Division, PINSTECH, Islamabad 44000, Pakistan

[g] MFMG, PD, PINSTECH, Islamabad 44000, Pakistan

* Corresponding Authors at: Nanomaterials Research Group, PD, PINSTECH, Islamabad 44000, Pakistan & State Key Laboratory of Chemical Resource Engineering, Beijing University of Chemical Technology, Beijing 100029, PR China.

Email Addresses: chempk@gmail.com (A. Nisar), mashkoorahmad2003@yahoo.com (M. Ahmad), xianggl@mail.buct.edu.cn (G. Xiang),

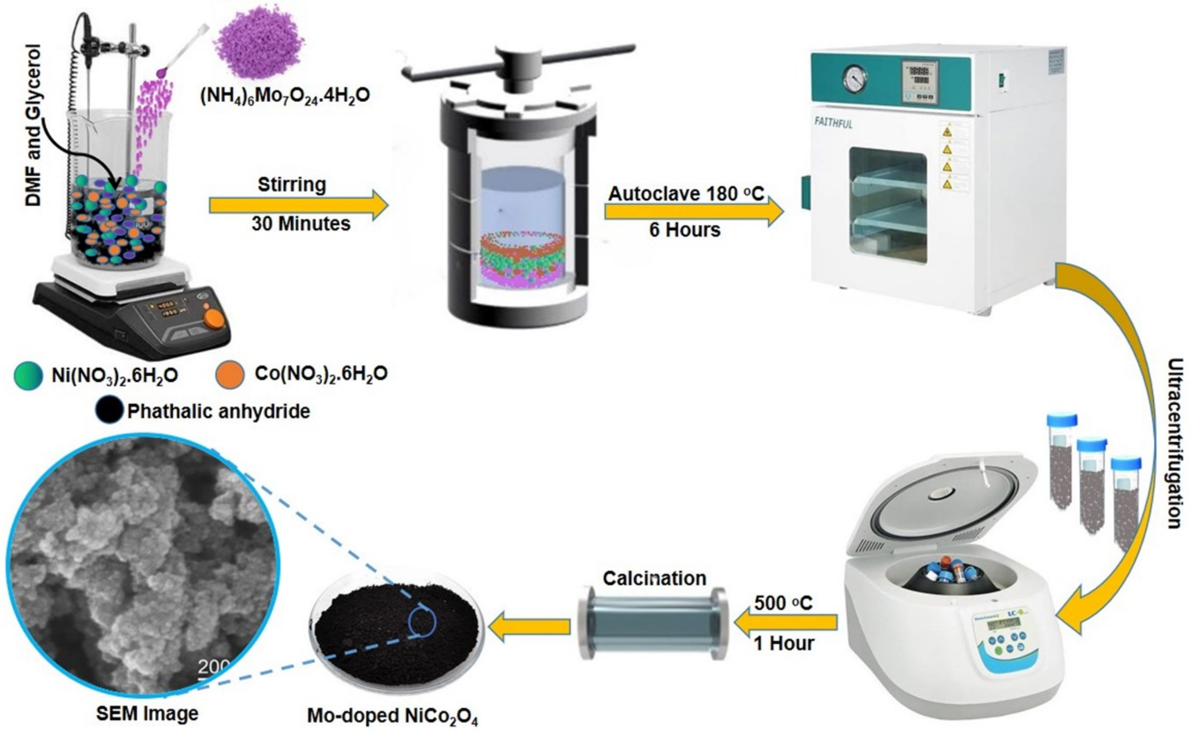

Fig. S1. Schematic illustration of synthesis of Mo-NCO nanostructures.

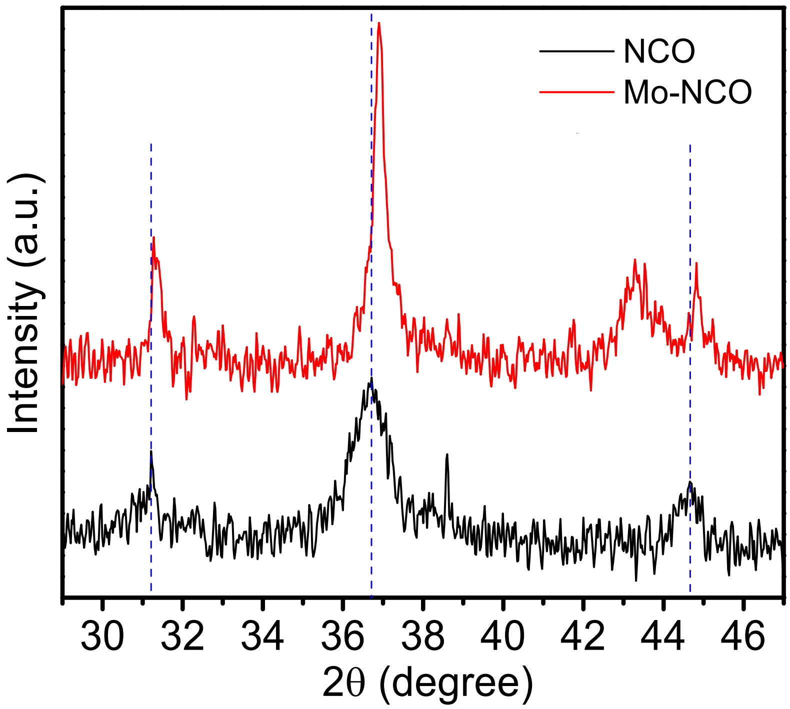

Fig. S2: XRD pattern of NCO and Mo-NCO nanostructures.

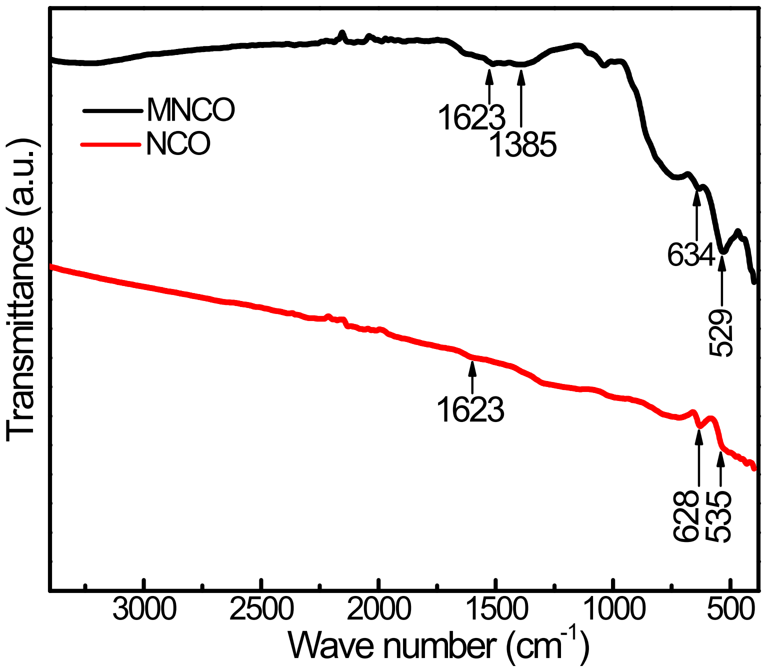

Fig. S3. FTIR spectra NCO and Mo-NCO nanostructures.

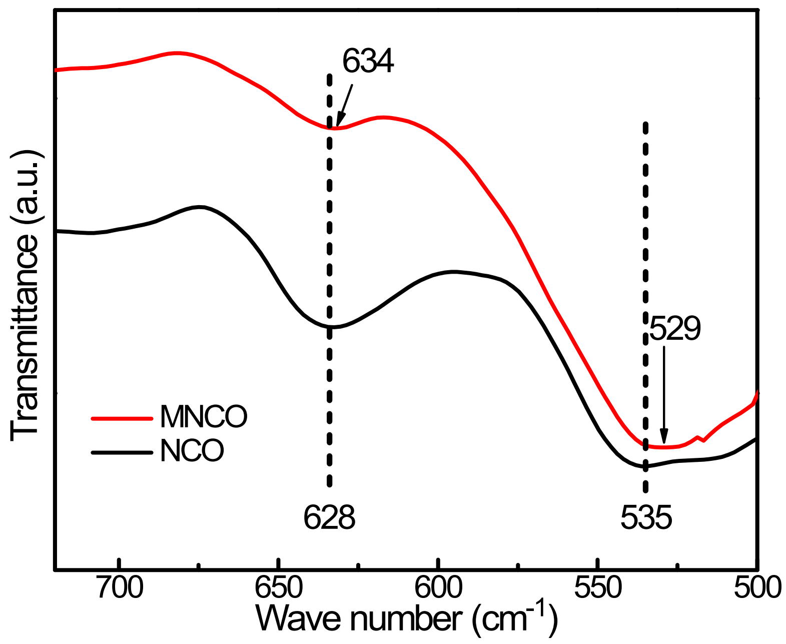

Fig. S4. Zoomed FTIR spectra NCO and Mo-NCO nanostructures showing the peak shift.

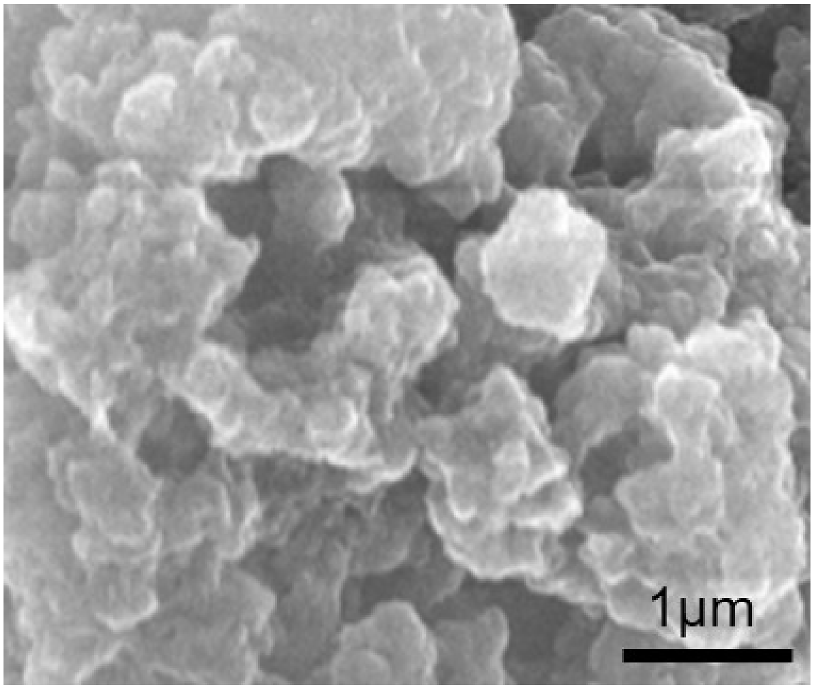

Fig. S5. SEM image of NCO nanostructures.

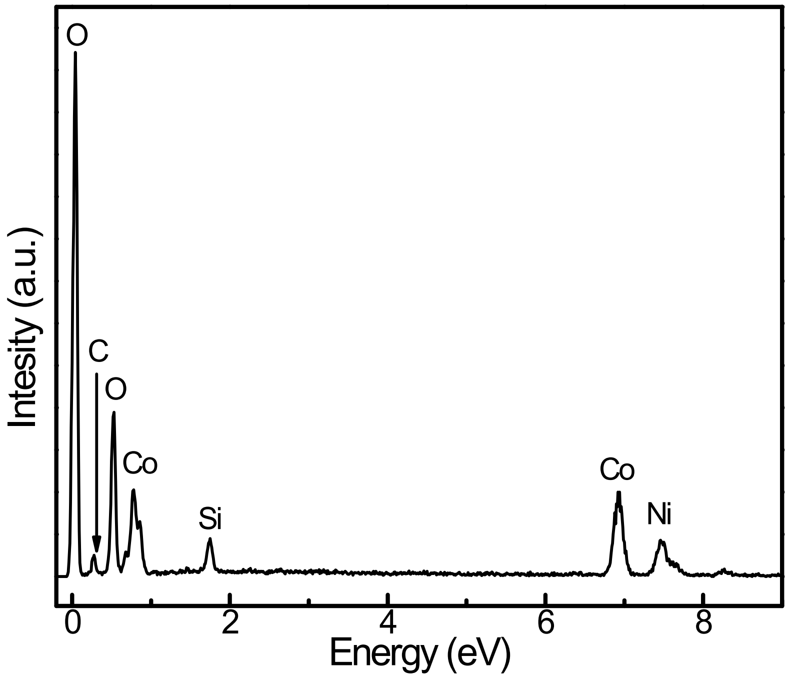

Fig. S6. EDX spectrum of NCO nanostructures.

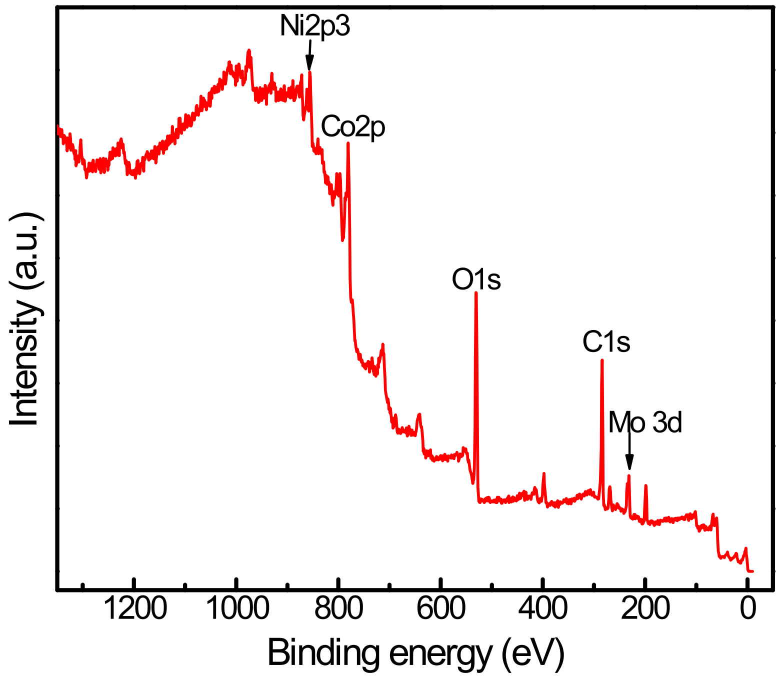

Fig. S7. XPS Survey spectrum of Mo-NCO nanostructures.

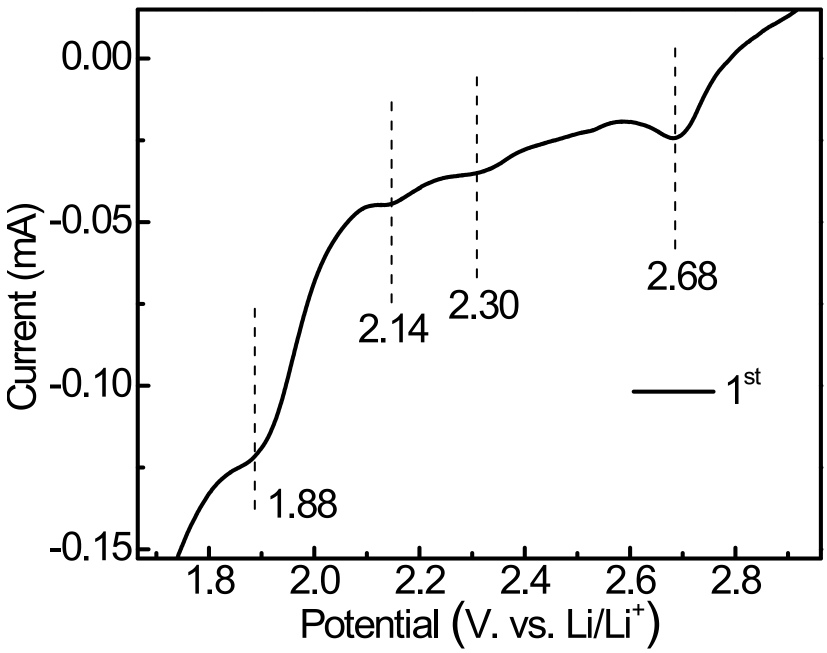

Fig. S8. Zoomed-in image of CV curve of 1st cycle of Mo-NCO electrode.

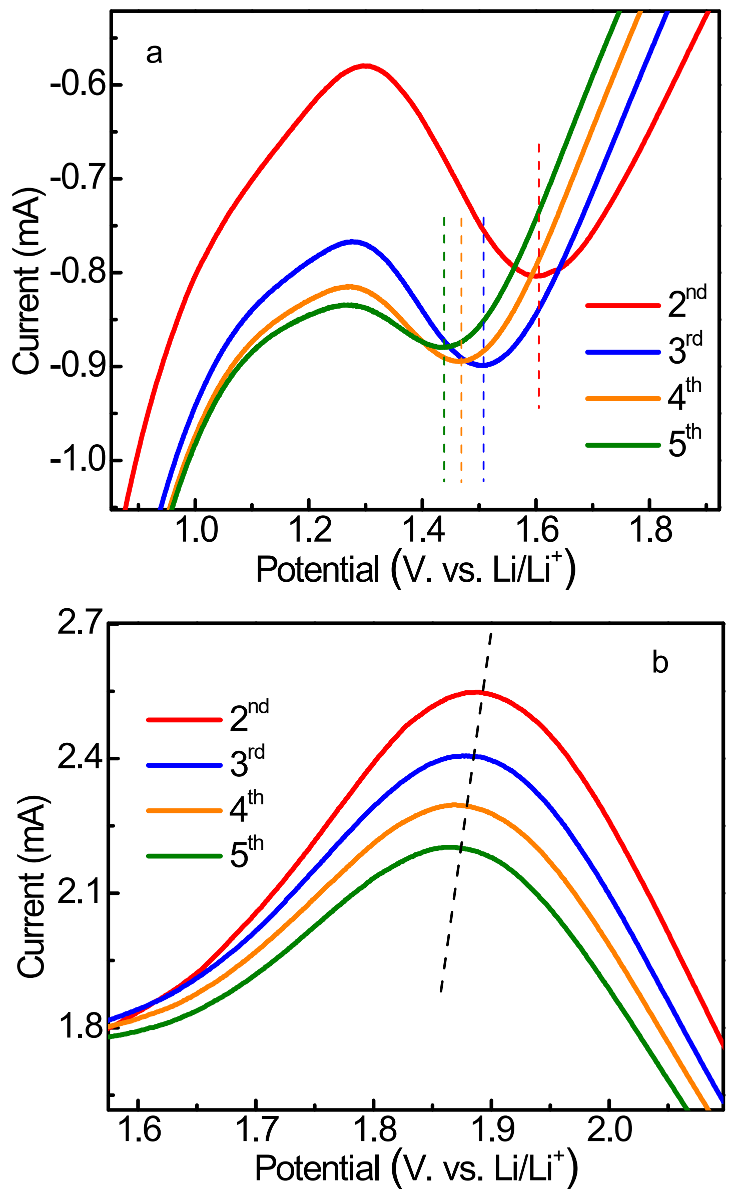

Fig. S9. Zoomed-in image of CV curves of 2nd, 3rd, 4th and 5th cycle Mo-NCO electrode. (a) Cathodic peaks. (b) Anodic peaks

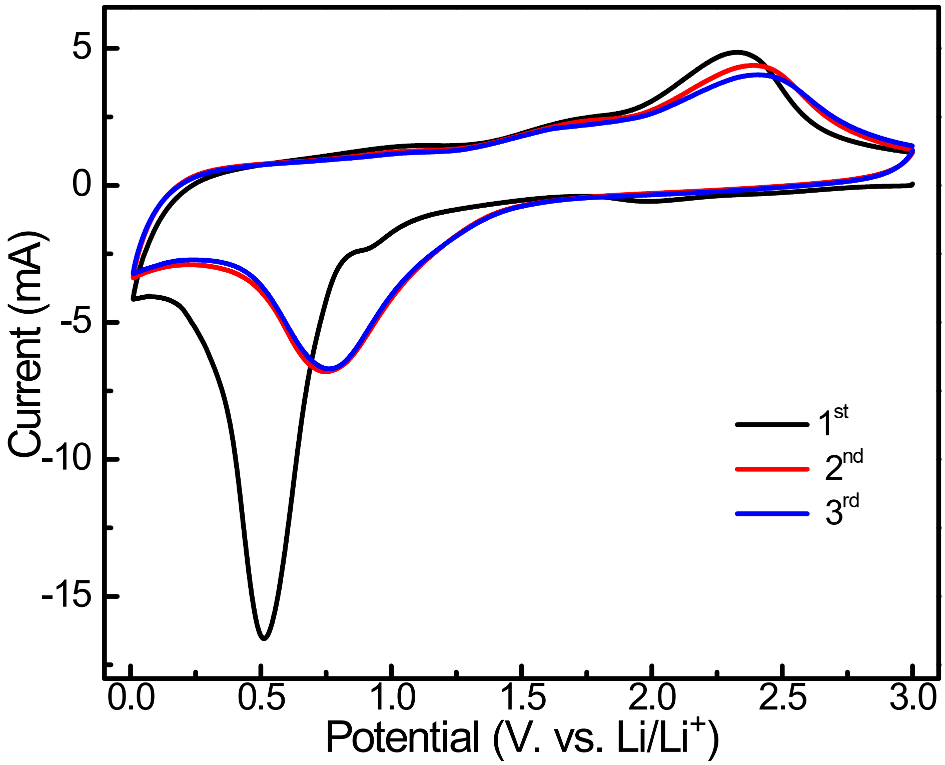

Fig. S10. CV curves of 1st, 2nd, 3rd cycles of NCO electrode at a scan rate of 0.5 mV s^{-1}.

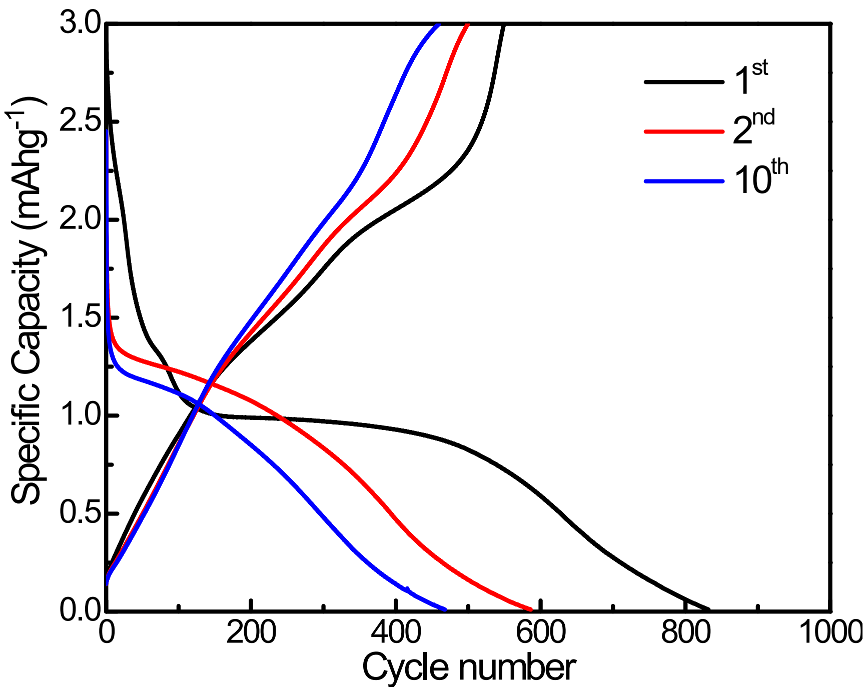

Fig. S11. Galvanostatic charge discharge profile of NCO electrode.

Table S1: Comparison of reversible capacity of $NiCo_2O_4$ with various materials.

**Material**	**Reversible capacity (mAh g^{-1})**	**Current density (mA g^{-1})**	**Number of cycles**	**Reference**
Mo-$NiCo_2O_4$	512	300	300	This work
NCO@Fe_2O_3	479	100	100	1
Pomelo peels-derived carbon (PPC)/ $NiCo_2O_4$	473.7	500	210	2
P-doped $NiCo_2O_4$	470	500	100	3
NCO@UNF	459	50	150	4
$NiCo_2O_4$/Ni	413	100	50	5
N-doped $NiCo_2O_4$	398	500	500	6
$NiCo_2O_4$/Al_2O_3	395	100	50	7
F-$NiCo_2O_4$@GO	387.4	400	100	8
$NiCo_2O_4$ microsphere	330.4	100	100	9
$NiCo_2O_4$ nanoplates	233	200	70	10

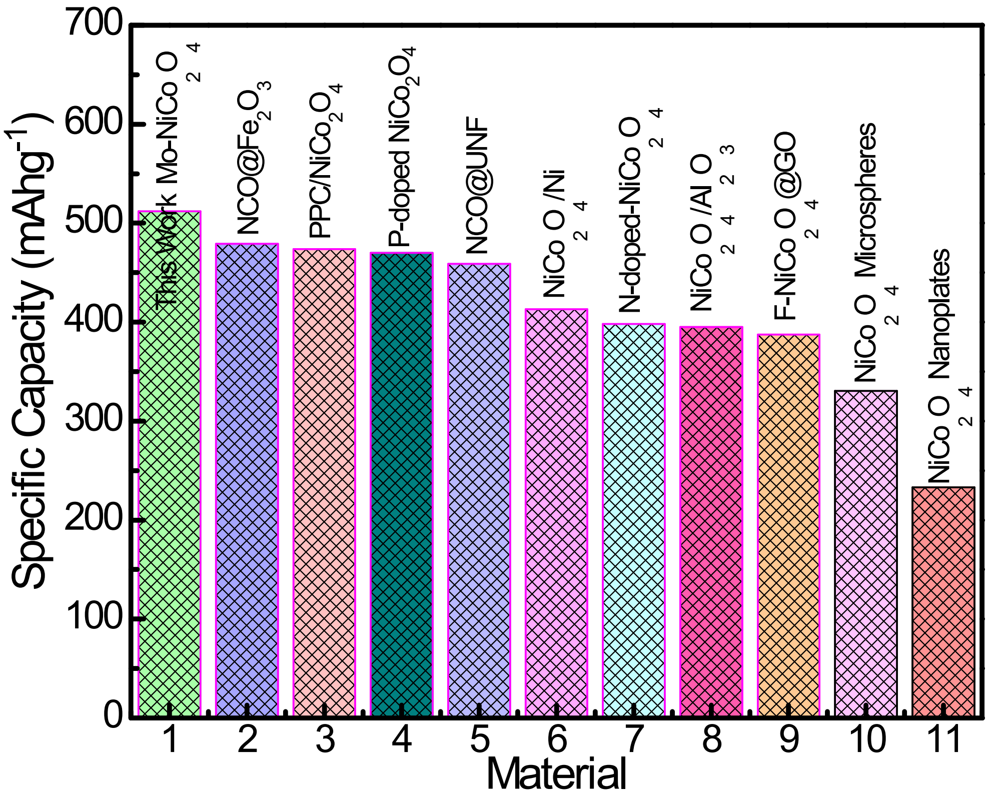

Fig. S12. Comperision graph of different $NiCo_2O_4$ materials with Mo-NCO electrode.

Table S2: Comparison of kinetic parameters of NCO and Mo-NCO electrodes

Sample	R_s (Ω)	R_{ct} (Ω)
$NiCo_2O_4$	6.5	193.3
Mo-$NiCo_2O_4$	2.4	103.5

To elucidate the electrochemical properties of Mo-doped $NiCo_2O_4$, the apparent Li-ion diffusion coefficient (D_{Li^+}) was determined from electrochemical impedance spectroscopy (EIS). The linear Warburg region observed in the EIS spectrum, characterized by a 45 degree inclination to the Z' axis, is ascribed to Li-ion diffusion within the bulk electrode material. The Warburg factor (σ) was subsequently calculated from the slope of the Z′ versus $\omega^{-1/2}$ plot within this Warburg region using equation (1):

$$Z' = R_1 + R_{ct} + \sigma\omega^{\left(-\frac{1}{2}\right)} \quad (1)$$

Figure S1(a,b) present the linear fit of *Z′* versus $\omega^{-½}$ in the low frequency region of Nyquist plots (Figure 4) of Mo-doped and pure $NiCo_2O_4$. The Li-ion diffusion coefficient (D_{Li^+}) is given by equation (2):

$$D_{Li^+} = R^2T^2/2A^2n^2F^4C^2\sigma^2 \quad (2)$$

Importantly, the apparent Li-ion diffusion coefficient (D_{Li^+}) is inversely proportional to the square of the Warburg factor (σ)."

Where R is the gas constant, T the absolute temperature, A the electrode surface area, n the charge transfer number during the redox process, F is the Faraday constant, C is the molar Li^+ concentration and σ the Warburg factor.

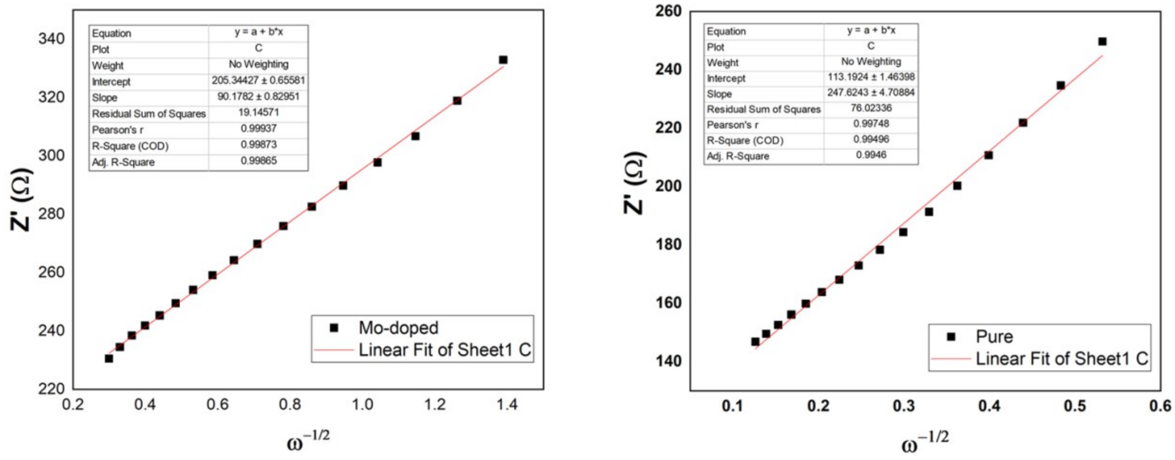

Fig. S13. Linear fit of Z' versus $\omega^{-1/2}$ in the low frequency region (Left) Mo-doped (Right) pure $NiCo_2O_4$

Referances

[1] Q.X. Chu, B. Yang, W. Wang, W.M. Tong, X.F. Wang, X.Y. Liu, J.H. Chen. Fabrication of a stainless-steel-mesh-supported hierarchical Fe_2O_3@$NiCo_2O_4$ coreshell tubular array anode for lithium-ion battery. *ChemistrySelect* **2016***, 1,* 5569.

[2] Y.D. Mo, Q. Ru, X. Song, J.F. Chen, X.H. Hou, S.J. Hu, L.Y. Guo, The design and synthesis of porous $NiCo_2O_4$ ellipsoids supported by flexile carbon nanotubes with enhanced lithium-storage properties for lithium-ion batteries. *RSC Adv.* **2016**, *6*, 31925.

[3] Zhang, C., Xie, Z., Yang, W., Liang, Y., Meng, D., He, X., ... & Zhang, Z. (2020). NiCo2O4/biomass-derived carbon composites as anode for high-performance lithium ion batteries. Journal of Power Sources, 451, 227761.

[4] Y.D. Mo, Q. Ru, X. Song, J.F. Chen, X.H. Hou, S.J. Hu, L.Y. Guo, The design and synthesis of porous NiCo2O4 ellipsoids supported by flexile carbon nanotubes with enhanced lithium-storage properties for lithium-ion batteries. RSC Adv. 2016, 6, 31925.

[5] G. H. Chen, J. Yang, J. J Tang and X. Y. Zhou, Hierarchical $NiCo_2O_4$ nanowire arrays on Ni foam as an anode for lithium-ion batteries, *RSC Adv.* **2015**, *5*, 23067.

[6] Jin, R., Yue, H., Xia, J., Ren, C., & Gao, S. (2021). Oxygen-Vacancy Abundant $NiCo_2O_4$ on the N-Doped Carbon Nanosheets as Anode for High Performance Lithium Ion Batteries. *ChemistrySelect* **2021**, *6*, 2029.

[7] Kou, H., Li, X., Shan, H., Fan, L., Yan, B., & Li, D. (2017). An optimized Al_2O_3 layer for enhancing the anode performance of $NiCo_2O_4$ nanosheets for sodium-ion batteries. *J. Mater. Chem. A* **2017**, **5**, 17881.

[8] Rong, H., Qin, Y., Jiang, Z., Jiang, Z. J., & Liu, M. (2018). A novel $NiCo_2O_4$@ GO hybrid composite with core-shell structure as high-performance anodes for lithium-ion batteries. **J. Alloys Compd. 2018**, *731*, 1095.

[9] K. Dong, Z. Wang, D. Wang, Morphological evolution of hollow $NiCo_2O_4$ microspheres and their high pseudocapacitance contribution for Li/Na-ion battery anodes. *New J. Chem.* **2018***, 42,* 17762.

[10] Chen, Y., M. Zhuo, J. Deng, Z. Xu, Q. Li and T. Wang, Reduced graphene oxide networks as an effective buffer matrix to improve the electrode performance of porous $NiCo_2O_4$ nanoplates for lithium-ion batteries. *J. Mater. Chem. A* **2014***, 2*, 4449.
